# 

**Published:** 1882-01

**Authors:** 


### INDEX

TO THE

### INDEPENDENT PRACTITIONER.

### VOLUME III.

### ORIGINAL COMMUNICATIONS.

**A**lcoholic Appetite, The. By Roger Marvin Griswold, M. D. .............. 366
Alcohol, The Physiological Action of. By John S. Lynch, M. D. .......... 73
Academy of Dental Science, The American. .............................. 569

**B**acteria the Cause of Caries. By F. Y. Clark, M. D., D. D. S. ............ 529

**C**roup and Diphtheria, Non-Identity of. By T. S. Lattimer, M. D. ........ 153

**D**ental Association, The American. ..................................... 510
Dental Society Meetings. By W. C. Barrett, M. D., D. D. S. ............. 436
Diphtheria, The Treatment of. By J. H. Carstens, M. D. ................. 289

**H**omœpathy. By A. B. Lyman, M. D. ...................................... 505

**I**ntrauterine Injections, The Advantages and the Dangers of. By Charles W. Pilgrim, M. D. ......................................................... 433

**L**arynx and Trachea, Obstructions in the. By E. Fletcher Ingals, M. D. ... 361
Laryngeal Phthisis. By J. D. Arnold, M. D. ............................... 217

**M**edicine and Surgery, The Unnecessary in. By S. V. Clevenger, M. D. ... 223

**P**ractice, An Incident of. By C. E. Francis, D.D.S., M.D.S. .............. 567

**S**hade Trees as a Sanitary Measure, Some Remarks on the Destruction of By Rufus W. Griswold, M. D. .....................................8, 79
Strabismus completed under Narcotism of the Bromide of Ethyl in fifty-two seconds for Ethylization and Tenotomy, Operation for. By Julian J. Chisolm, M. D ........................................................ 625

**T**ampon in the Hemorrhage of Abortion, Some Observations on the Use of the. By Rufus W. Griswold, M. D. ..................................... 629

**U**rethrotomy With Cases, External. By Charles F. Bevan, M. D. .......... 1

**W**inter Cold, A. By F. H. Bosworth, M. D. ........................ 561, 593

### CLINICAL REPORTS.

**A**cute Lobar Pneumonia with Cardiac Failure. By Charles W. Pilgrim, M.D. 292
Aneurism of the Innominate Artery. By J. S. Lynch, M. D. .............. 295

**C**ancer of the Left Mammary Gland, followed by Tetanus I. Cancer of the Antrum, II. By Charles B. Zeigler, M. D. .......... 375
Case of Complete Intestinal Obstruction Caused by the Appendix Vermiformis in the Femoral Canal; Operation and Recovery .......... 442

**G**lobe Pessary Worn 35 Years Without Removal, A. By A. Reeves Jackson, A. M., M. D. .......... 83

**I**nferior Maxillary Nerve in the Spheno-Maxillary Fossa, A New Operation for Exsection of the .......... 11

**P**aralysis, Facial. By Jas. T. Whittaker, M. D. .......... 145
Philadelphia Hospital of Oral Surgery, Four Cases in the. By Jas. E. Garretson, M. D. .......... 225

**R**ecovery after Taking and Retaining Two Hundred and Eighty Grains of Chloral and Two Grains of Morphia. By R. M. Griswold, M. D. .......... 84

Syphilis of the Finger in Medical Men, Eight Cases of. By Fessenden N. Otis, M. D. .......... 158

CORRECTION .......... 233

CORRESPONDENCE .......... 163, 299, 378

ORIGINAL TRANSLATIONS AND ABSTRACTS.

**A**nkle Joint Disease, The Prognosis and Treatment of .......... 300
Antiseptic Solutions to be Used in Surgery, The Proper Strength of .......... 455
Antrum, Treatment of Catarrh of the .......... 380

**B**acillus Lepræ? Is there a .......... 304
Buccal Ulcerations of Constitutional Origin .......... 174

**C**arcinoma of the Breast, The Influence of Operations upon the Prolongation of Life and Permanent Recovery in .......... 308
Chancres of the Cervix Uteri, The Characteristics of .......... 233
Clavical by Suturing the Fractured Ends, Treatment of Fracture of the .......... 236

**D**elirium Produced by Salicylic Acid .......... 314
Dental Science, The American Academy of .......... 569
Diabetic and Nephritic Neuralgias, On .......... 379
Dyptheritic Cystitis .......... 538

**E**ar Disease, The Use of Boracic Acid and Calendula in .......... 90
Ergot, Physiological and Therapeutical Action of .......... 235
Ergot in the Treatment of Glycosuria .......... 568
Ether, The Subcutaneous Injection of .......... 569
Eustachian Tube, The Treatment of Inflammation of the .......... 383
Eye Diseases, Value of Jaborandi and Pilocarpine .......... 164
Eye Protector for use with the Monocular Microscope, An .......... 454

**H**erpes Zoster and Muscular Atrophy, Combination of .......... 381
**I**ntracparial Disease and Choked Disc .......... 383
Intussusception, The Treatment of .......... 540

**K**idney, The Epithelia of the .......... 389

Laryngo-tracheal Phthisis, The Pathological Anatomy of.................. 455
Lethurgy, Recurring.................................................. 232

Mercury as Used in Amalgam Fillings, The Chemistry and Physiological Action of............................................................ 237

Nerve Stretching, Recent Cases of...................................... 93
Nerve, Stretching the Optic.......................................... 234
Neuralgia, The Treatment of Certain Forms of........................... 92

Otorrhœa, Borax and Boracic Acid in................................. 314
Ovarian Cell Pathognomonic? Is the................................... 313

Pericementitis ....................................................... 450
Pneumonia, The Geographical and Climatic Relations of................. 453

Quinine in Gynecic and Obstetric Practice............................. 21

Saliva in Syphilitics, Characteristics of ............................ 315
Salivary Glands, The Innervation of the................................ 23
Skin, The Influence of the Nervous System upon Pathological Changes in the 382
Spinal Irritation...................................................... 86
Surgery and the Doctrine of Evolution................................ 539
Surgery of the Pericardium, The........................................ 12
Syphilis and Locomotor Ataxia........................................ 444
Syphilis, Early Joint Affections in.................................. 537

Teeth and Alveolar Portions of the Jaws, Investigation into the Effects of Organisms upon the.............................................. 167
Teeth, The Action of Ammonia on the.................................. 385

Urine in Elderly Men, Retention of..................................... 93
Uterine Appendages for the Arrest of Uterine Hemorrhage, Removal of the. 89
Uterus, A New (Bloodless) Method of Removing Subserous Fibroids of the. 173

Vaccination with Animal and Human Virus............................... 17
Vicarious Menstruation............................................... 314

Wounds, The Modern Treatment of........................................ 15

### SELECTIONS.

Abortion as a Therapeutic Measure, On the Induction of............... 105
Accidental Ante-Partum Hemorrhage...................................... 24
Aconite in Dysentery................................................. 579
Aconite Poisoning.................................................... 188
Aconitines, The Physiological Action of Commercial................... 400
Albuminuria and Eclampsia During Pregnancy........................... 477
Albuminuria, Significance of......................................... 483
Anti-Vaccinationists, One of the Vagaries of the..................... 639
Artificial Hunyadi Janos Water....................................... 184
Arsenical Paralysis.................................................. 332
Artificial Tetanus, The Causes of Death in............................ 42
Aspiration of the Bowels in Peritonitis.............................. 185

**B**acteria, The Latest About.......... 638
Blood-Letting, The Physiological Action of.......... 527
Brain, A Heavy.......... 646
Brain, A Remarkable Wound of the.......... 471
Brains of Animals, Recent Results of Experiments Upon the.......... 107
Bromide of Sodium and Epilepsy.......... 476

**C**ancer, The Pre-Cancerous Stage of.......... 464
Carbolic Acid in Urine.......... 526
Carbolized Catgut Sutures for Lacerations of the Cervix Uteri.......... 258
Catarrhal Ozœna.......... 614
Cavities in the Lungs, Opening and Drainage of.......... 116
Cerebral Excitement Caused by the Bromides.......... 181
Cerebral Pathology.......... 114
Chancres of the Preputial Margin, On Simple.......... 457
Chloroform, Deaths from.......... 263
Chloroform, Impure.......... 249
Chloroforming Mice.......... 547
Chloroform, Action of.......... 396
Chorea, Arsenical Treatment of.......... 477
Chronic General Peritonitis.......... 187
Columbus Medical College.......... 544
Communicable Diseases, Periods of Incubation of.......... 40
Constipation in Females.......... 644
Constipation in Infants.......... 330
Contagious Peripneumonia and Preventive Inoculation.......... 190
Crural Neuralgia Among Dentists.......... 572
Cyst of the Pancreas.......... 329

**D**amiana—Unexpected Results from Its Use in a Case.......... 610
Delirium Tremens.......... 475
Dental Literature, Remarks of Dr. W. C. Barrett Upon.......... 575
Dental Science, American Academy of.......... 35
Dental Surgeons in the Army and Navy.......... 259
Dentistry, A Triumph of.......... 261
Dentition, The Disorders of Primary.......... 402
Diagnosis of Fœtal Monstrosities.......... 644
Digitalis, Indications for the Administration of.......... 121
Diptheria.......... 640
Diphtheria, Some Facts About.......... 186
Dysmenorrhea.......... 605

**E**cxema of the Fingers and its Treatment, On.......... 96
Electrotonus of Human Nerves, Waller and DeWatteville on the.......... 472
Embalming Bodies and Preserving Tissues, A New Method of.......... 182
Emulsions, and How to Make Them.......... 320
Epilepsy, The Pathology of.......... 40
"Erysipelas" of the Face, Treatment of Frequently Recurring.......... 190
Erysipelas, The Prophylaxis of.......... 329
"Ethereal" Castor Oil.......... 189

Exposed Pulps, A New and Original Method of Treating.................. 36
Eye from Affections of the Teeth, Cases of Diseases of the................. 265
Filaria Sanguinis Hominis........................................ 333
Fingers, Dupuytren's Contraction of the.... ............................. 469
Fistula in Ano, Hints in Operating for............ .................. . 176
Foot, Pain in the................................................ 246
Fractures, The Use of Plaster of Paris in.............................. 180
Freezing Cure, The.................. ........................... 47
Frozen Animals, Resuscitation of............................. ............. 187
Gallstones Passed by an Infant........................................ 464
General Surgery in which the Dental Engine was Employed for Exsection of Bone, Three Cases in.................... ....................... 111
Genital Irritation.......................................... ............ 407
Goitre, The Causes and Treatment of................................ 45
Gonorrhea, The Positive Treatment of.................................. 608
Guachamaca Extract.... ......................................... 574
Hard Palate and Hare-Lip immediately after Birth, Abstract of Paper on Operation for Closure of the........................................ 570
Headaches in Children........................................... 408
Health of Criminal Women........................................... 641
Helleborein—A New Heart Stimulant................................... 119
Heredity of Sebaceous Cysts......................... ................ 651
Hernias of Long Standing, Treatment of................................. 330
Human Teeth, Civilization in its Relation to the Increasing Degeneracy of.. 27
Hydatids of the Lungs, A Case of......................................... 603
Hypodermic Injection, Improvements in.................................. 189
Hysteria.................................................................. 262
Impotency.................. .............................................. 580
Insanity, Moral................................................... 332
Iodide of Potassium in Frontal Headache...................... .......... 260
Kidney, The Surgery of the........................................... 397
Kidneys for Nephro-Lithiasis, Removal of the.............................. 184
Knee, Excision of............................................................ 265
Lactation, Effect of Drugs on ....... ..................................... 189
Larnyx, Extirpation of the ................................................ 47
Leucorrhœa of Pregnancy.............................................. 103
Locomotor Ataxia, The Electric Brush in the Treatment of................ 479
London, The Health of........ ................................... 546
Male Sexual Hermicrania ............................................. 601
Medical Museum and Library, The Army........................ ........ 637
Medico-Legal Decision, An Important.......................... ....... 647
Maternal Impressions..................................... ....... 265
Metallic Cap-Filling.................................. ................. 37
Metallotherapy.............................................................. 30
Microbe of Typhoid Fever, On the....................... ... .......... 634
Multiple Cerebro-Spinal Sclerosis..................................... 470
Myxo-Angioma of the Skin, Studies on.................................... 612

**N**asal Catarrh, Treatment and Cure of.......... 398
Nerve Elements in Whooping Cough, The.......... 474

**O**leoresin of Male Fern : Increasing in Efficacy against Tapeworm.......... 573
Opium, Giving.......... 190
Opium Habit, Its Successful Treatment by the Avena Sativa?.......... 580
Opthalmia Neonatorum, Nitrate of Silver to Prevent.......... 615
Optico-Cillary Neurotomy, A Simple Way of Performing—The Proposed Substitute for Enucleation.......... 327
Osseous Lesions in Hemiplegia.......... 191
Osseous Tissues Formed from Transplanted Bone-Marrow.......... 335
Otalgia from Reflex Dental Irritation.......... 541
Ovaries, The Position of the.......... 468
Ovary, Hernia of the.......... 466
Ovum, Migrations of the.......... 407

**P**arasite, The Tubercle—A New Theory of Consumption.......... 389
Paracentesis Cranii in Cases of Hydrocephalus.......... 482
Paris Academy of Medicine, The Sixteen Commandments of the.......... 101
Papillary Growths of the Leg Preceding Cancer.......... 480
Pathology of Daltonism, The.......... 43
Pepsin, A Novel Use for.......... 642
Permanent Removal of Hair by Electrolysis, The.......... 245
Pilocarpine in Edema of the Glottis, Injections of.......... 193
Pilocarpine, The Action and Uses of.......... 178
Political Myopia.......... 264
Potassium Bromide in Orchitis.......... 250
Pregnant Women, Extracting of Teeth of.......... 578
Prophylaxis of Venerial Diseases.......... 262
Proprietary Medicines.......... 580
Puerperal Convulsions.......... 107
Puerperal Eclampsia, Hot Pack in.......... 615
Puerperal Infection in the Male.......... 42
Pyrogallic Acid in Soft Chancre.......... 48

**R**eckless Midwifery.......... 646
Rheumatismal, Pott's Disease.......... 473

**S**aline Arterial Injections ia Collaose.......... 331
Scarlet Fever, Infectivity of.......... 395
Scrofulous Joint Inflammations.......... 104
Scrotal Calculus.......... 192
Sick Headache.......... 607
Skin-Grafting, Our Present Knowledge.......... 336
Slaughter House Examinations.......... 545
Smallpox, Look out for.......... 644
Smoke Nuisance, The.......... 546
Some Experiments on the Action of Organic Matters upon Silver Salts.......... 399
South Carolina State Board of Health Proposes to Accomplish, What the.... 251
Strychnia Poisoning in a Dog cured by Chloral Hydrate.......... 26
Subcutaneous Section of Adhesions for Reduction of Old Dislocations of the Shoulder Joint.......... 485

Successful Midwifery—Baby Incubator.............................. 615
Suppuration without Micro-Organisms.............................. 261
Sulphurous Acid as a Disinfectant.............................. 485
Swallowing Artificial Teeth.............................. 645
Syphilitic Chancre, Effects of Excision of.............................. 192
Syphilis, Mercury and other Remedies in the Treatment of.............................. 328

**T**etanus, Anatomical Basis of.............................. 614
Thirst, The Sensation of.............................. 334
Tincture of Burdock in Psoriasis Inveterata.............................. 642
Tinea Versicolor in a Child.............................. 469
Tobacco-Chewing Boys take Warning.—Undeveloped Testes Associated with Early Tobacco-Chewing.............................. 646
Transient Albuminuria as it Occurs, Particularly in Children and Adolescents, in Apparent Health.............................. 117
Traumatic Tetanus treated with Eserine.............................. 579
Trephining the Skull, A New Method of.............................. 252
Tuberculosis and Scrofulosis, Relations of.............................. 484
Tuberculosis, The Micrococci of.............................. 37
Tympanites, Treatment of.............................. 193
Typhoid Fever, The Antiseptic Treatment of..............................545, 606
Typhoid Fever Epidemic in Paris.............................. 614

**U**remia, Experimental.............................. 115
Uremia in Children, Treatment of by Pilocarpin.............................. 473
Urinary Bladder, Partial Resection of the.............................. 481
Uterine Diseases.............................. 183

**V**accinal Skin Eruptions.............................. 392
Vaccination, Some Points Concerning.............................. 254
Vaginal Hernia, Spontaneous Cure of.............................. 261
Veneral Diseases, Prophylaxis of.............................. 262
Viburnum Prunifolium.............................. 642
Vicarious Menstruaion, Case of.............................. 482
Virgins, Uterine Hydatids in.............................. 471

**W**arts, On the Removal of.............................. 122
Wartz, Venereal.............................. 188
What is Tubercle ?.............................. 324
When Does the Danger of Infection in Scarlatina Cease ?.............................. 193

**Y**ellow Fever, Prof. Pasteur and the.............................. 186

### EDITORIAL DEPARTMENT.

**A**mbulance System, The New York.............................. 124
American Medical Association, The Publication of the Transactions of the.. 486
American Medical Association, The.............................. 487
American Medical Association, The Exclusion of the New York Delegates from the.............................. 409
American Public Health Association, The..............................52, 125

Apology, An.... 528
April Issue, Delay of the.... 270
Are we Slaves or Freemen?.... 340
August Issue, In our.... 549

**B**acteria.... 548
Baltimore College of Dental Surgery, The.... 410

**C**ollaborators, Our.... 122
Correction.... 123
Curiosities of Heredity, The.... 270

**D**ental Department of University of Maryland.... 269
Dental Education Abroad and at Home.... 122
"Dental Society Meetings".... 488
Dental Exchanges, Articles for Publication and Books for Review in the Dental Department.... 649
Dentistry, New York College of.... 411
Dentists, The Higher Professional Duties of.... 123

**E**ditor on His Rambles, The Senior.... 196
Ethics of the New York State Medical Society and the American Medical Association, The Code of.... 338

"**H**eated Terms," The Excessive.... 488
Hospital Saturday and Sunday.... 51

**I**ndependent Practitioner Co., Dissolution of the.... 649
Is Deafness Increasing?.... 341

**M**edical Ethics, A Chapter on.... 266
Medical Exchanges, Articles for Publication, and Books for Review in the Medical Department.... 649

**P**hysicians During Epidemics, The Protection of.... 49
Physiological Experiment in England, The Crusade against.... 194
Popular Science Exchanges and Books.... 649
Post-Graduate Faculty of the Medical Department of the University of the City of New York, Resignation of the.... 341
Professional Education.... 548
Publisher's Notice.... 650

**S**eptember, October and November Issues, Our.... 548
Small-Pox Decreasing in New York.... 53
Some False Impressions Corrected.... 410

**T**rade-Mark Controversy Again, The.... 549
Tuberculosis, The Alleged Discovery of the Cause of.... 409

"**U**nsavory Journalism".... 616

**V**aledictory.... 648
Volume, Our New.... 49

**C**URRENT NEWS AND OPINION. 53-56, 126-130, 197-198, 270-272, 342-345, 411-414, 489-491, 550-551, 582-583, 616-617.

OBITUARY........................................................................ 199

REVIEWS AND BIBLIOGRAPRICAL NOTICES..57-59, 131-133, 200-202, 272-275, 346-348, 414-416, 491-494, 552-553, 583-586.

POPULAR SCIENCE DEPARTMENT.

American Invention in the Egyptian War........................... 556
Ancients for Animals, Regard of the................................ 654

Baboons............................................................ 284
Beetles, Some Ground............................................... 349
Birth and Breeding.............................................276, 355
Burns and Scalds, Soda in.......................................... 282

Cell Can Do, What the.............................................. 633
Census of Great Britain, The....................................... 503
Chemical Difference Between Living and Dead Protoplasm............. 287
Chin as an Index of Character, The................................. 555
Cliff Dwellers, A Discovery of a City of........................... 349
Clouds? What are................................................... 559
Coal, A New Theory of the Formation of............................. 427
College Lodging House Supervision.................................. 142
Colorado Desert, The............................................... 421
Copying Ink Without a Press........................................ 214

Dentition in the Horse and Other Animals........................... 495
Drams, Morning..................................................... 351
Dusts, Dangerous Properties of..................................... 416

Earthquakes, Ancient Views Concerning.............................. 420
Earthquakes in 1881................................................ 501
Electric Discharge, Forms and Functions of the..................... 210
Electric Horticulture.............................................. 421
Electric Light Wires, Accident with................................ 557
Electric Lightning, Real Workers in................................ 143

Fishy Curiosities.................................................. 499

Gas, The Future of................................................. 620
Gastroscope, The................................................... 142
Gout and an Animal Diet............................................ 215
Grand Hall near the Pantheon, at Rome, A Discovery of a............ 560
Greenland Glaciers, The............................................ 353
Gun Camera, Using a................................................ 424

India-Rubber Ring on a Mackerel.................................... 141
Industrial Education in the Public Schools......................... 558
International Congress of Hygiene at Geneva, The................... 586
Is it Worth Domesticating?......................................... 420

Lyell's Ancestry, Sir Charles...................................... 348

**M**astodon Skeleton Found ... 621
Military Ants of the Amazon ... 68
Monkeys ... 283
Music, The Origin ... 617

**N**itro-Glycerine, Properties ... 554

**P**arasitic Fungi, Acti on of ... 427
People in Russia, A New Race of ... 502
Plant Longevity ... 286
Photo-Printing in Colors ... 352
Photometer, A Pocket ... 589
Porcelain, The Discovery of ... 500
Preservative Compound, A New ... 424
Problem, A Curious ... 422

**S**andias--Past and Present, The ... 352
Sanitation, The Relative Importance of Various Subjects in Practical ... 134, 203
Scientific Smuggler, A ... 354
Sewer-Gas and Disease, Dr. Barker on ... 588
"Smartness" of Worms and Fish, The ... 423
Solar Energy ... 287
Soups and Broths, Virchow on ... 428
Spectroscope Foretells Rain, How the ... 622
Stars, The Light of ... 71
Steel Spring, A Monster ... 623
Suicide Statistics ... 430
Sun, Annular Eclipse of the ... 618

**T**eeth, Civilization and Decay of the ... 71
Tuberoses, The Culture of ... 139

**V**egetables in Winter, Keeping ... 212
Violets, A Plague Among the ... 70

**W**ar Handkerchiefs ... 426
Water, The Blue Color of ... 428
Women, A New Profession for ... 590
Worms, The Work of ... 59

VOL. III. JANU[illegible]Y, 1882. NO. I.

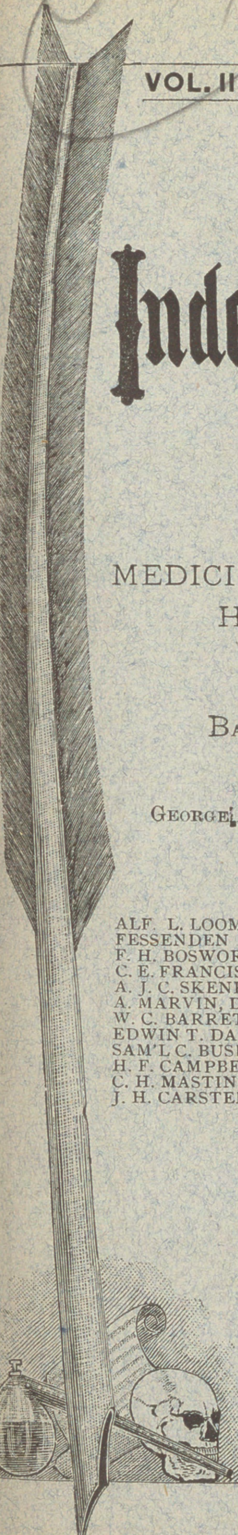

THE

### Independent Practitioner:

A MONTHLY JOURNAL,

DEVOTED TO

MEDICINE, SURGERY, OBSTETRICS, DENTISTRY, HYGIENE AND POPULAR SCIENCE.

EDITORIAL CORPS:

BASIL M. WILKERSON, D.D.S., M.D., EDITOR.

Associates:

GEORGE H. ROHÉ, M. D. GEO. W. FIELD, D. D. S.

COLLABORATORS:

ALF. L. LOOMIS, M. D... ........New York.
FESSENDEN N. OTIS, M. D..... "
F. H. BOSWORTH, M. D.......... "
C. E. FRANCIS, D.D.S., M.D S..... "
A. J. C. SKENE, M. D........ Brooklyn, N. Y.
A. MARVIN, D. D. S.......... " "
W. C. BARRETT, M. D., D. D.S.. Buffalo, "
EDWIN T. DARBY, M.D., D.D.S... Phil., Pa.
SAM'L C. BUSEY, M. D.,........Wash'n, D. C.
H. F. CAMPBELL, M. D.........Augusta, Ga.
C. H. MASTIN, M. D., L.L.D......Mobile, Ala.
J. H. CARSTENS, M.D..........Detroit, Mich.
J. J. CHISOLM, M. D.. ........Baltimore, Md.
C. F. BEVAN, M. D............ " "
I. E. ATKINSON, M. D.......... " "
H. McGUIRE, M. D............Richmond, Va.
F. P. PORCHER, M. D.,........Charleston, S.C.
J. W. UNDERHILL, M. D..... Cincinnati, O
JAS. T. WHITTAKER, M. D.. " "
J. RANSOHOFF, M.D., F.R.C.S.. " "
F. FORCHHEIMER, M. D....... " "
A. R. JACKSON, M. D.......... Chicago, Ill.
S. V. CLEVENGER, M. D........ " "
E. F. INGALS, M. D............ " "
PHILIP S. WALES, M. D., Surg. Gen'l, U. S. Navy.

NEW YORK:

*THE INDEPENDENT PRACTITIONER CO.,*

1099 BROADWAY.

$3.00 Per Annum in Advance. - - - - Single Copies 30 Cents.

*Entered at the Post Office, New York City, as Second-Class Matter.*

WILL *YOU* PLEASE SEND IN *YOUR* SUBSCRIPTION MONEY *NOW?*

### Personal.

*This copy of the INDEPENDENT PRACTITIONER is sent to you with the hope that you will subscribe.*

*It will not be continued to any one, except our old PAID UP Subscribers, UNLESS so ordered.*

*Please let us hear from you, and greatly oblige*

*Yours faithfully,*

The Independent Practitioner Co.,

1099 BROADWAY, N. Y.

*This Column For Sale to a First-Class Advertiser.*

**Money is at Our Risk Only when sent by Post Office Money Order, Registered Letter, or Check made payable to the Independent Practitioner Co., 1099 Broadway, N. Y. Please add 25 Cents for collecting checks payable outside of New York City.**

